# Recruitment of Factor H to the *Streptococcus suis* Cell Surface is Multifactorial

**DOI:** 10.3390/pathogens5030047

**Published:** 2016-07-07

**Authors:** David Roy, Daniel Grenier, Mariela Segura, Annabelle Mathieu-Denoncourt, Marcelo Gottschalk

**Affiliations:** 1 Research Group on Infectious Diseases in Production Animals and Swine and Poultry Infectious Diseases Research Centre, Faculty of Veterinary Medicine, University of Montreal, Saint-Hyacinthe, J2S 7C6, Quebec, Canada; david.roy.6@umontreal.ca (D.R.); mariela.segura@umontreal.ca (M.S.); annabelle.mathieu-denoncourt@umontreal.ca (A.M.-D.); 2 Oral Ecology Research Group, Faculty of Dentistry, Laval University, Quebec City, J2S 7C6, Quebec, Canada; Daniel.Grenier@greb.ulaval.ca

**Keywords:** *Streptococcus suis*, factor H, factor H-binding proteins, adhesion, polysaccharide capsule, phagocytosis, C3b, complement

## Abstract

*Streptococcus suis* is an important bacterial swine pathogen and a zoonotic agent. Recently, two surface proteins of *S. suis*, Fhb and Fhbp, have been described for their capacity to bind factor H—a soluble complement regulatory protein that protects host cells from complement-mediated damages. Results obtained in this study showed an important role of host factor H in the adhesion of *S. suis* to epithelial and endothelial cells. Both Fhb and Fhbp play, to a certain extent, a role in such increased factor H-dependent adhesion. The capsular polysaccharide (CPS) of *S. suis*, independently of the presence of its sialic acid moiety, was also shown to be involved in the recruitment of factor H. However, a triple mutant lacking Fhb, Fhbp and CPS was still able to recruit factor H resulting in the degradation of C3b in the presence of factor I. In the presence of complement factors, the double mutant lacking Fhb and Fhbp was similarly phagocytosed by human macrophages and killed by pig blood when compared to the wild-type strain. In conclusion, this study suggests that recruitment of factor H to the *S. suis* cell surface is multifactorial and redundant.

## 1. Introduction

Infections caused by *Streptococcus suis* represent an economic problem for the swine industry, being one the most important bacterial pathogen for weaned piglets [1]. It usually causes septicemia with sudden death, meningitis, arthritis, endocarditis and other infections [1]. Moreover, *S. suis* is a zoonotic agent causing meningitis, septicemia and toxic shock-like syndrome in humans [2]. In Western countries, human *S. suis* infections are mainly associated with the swine industry, whereas, in some Asian countries, the general population is at risk and *S. suis* is one of the main causes of adult meningitis [3]. Serotype 2 is the most virulent and the most commonly isolated serotype in swine and humans [4]. The pathogenesis of the *S. suis* infection is only partially known; it has been proposed that, in swine, bacteria first colonize epithelial cells of the respiratory tract, and then reach the bloodstream where they can survive and multiply [5]. The capsular polysaccharide (CPS), rich in sialic acid, plays an important antiphagocytic role [5]. In addition, *S. suis* possesses different surface proteins implicated in host colonization and resistance to host immune response. While several of these surface proteins have already been characterized, many remain poorly studied and their physiological and pathological roles uncharacterized [5].

Factor H is a soluble complement regulatory protein that protects host cells from complement-mediated damages [6]. This plasma glycoprotein is the key fluid phase regulator of the alternative complement pathway and acts as a cofactor in the factor I-mediated proteolysis of C3b. Proteolytic cleavage of C3b results in the formation of the inactive iC3b fragment, which remains covalently linked to the surface [7]. It also competes with factor B for binding to C3b, therefore interfering with the formation of the C3bBb complex [7]. However, many bacterial pathogens have the ability to bind factor H to their cell surface in order to avoid complement attack and opsonophagocytosis. In several bacterial pathogens, surface-exposed proteins and/or sialic acid-rich polysaccharide components have been shown to be able to bind factor H [8]. In addition to its complement regulatory function—which may modulate opsonophagocytosis—factor H has been shown to contribute to bacterial adherence to different host cells [9].

It is known that *S. suis* is able to bind factor H as a cofactor in order to degrade C3b in the presence of complement factor I [10]. Two different surface proteins of *S. suis*, Fhb and Fhbp, have so far been described for their capacity to bind factor H [10,11]. Recombinant Fhb and Fhbp proteins have a molecular weight of 130 kDa, and 95 kDa, respectively [10,11]. Although both proteins share low protein identity (33%), they possess a LPXTG sequence followed by hydrophobic domains recognized by the sortase A anchoring enzyme [10,11]. Thus far, only Fhb was characterized for its role in the pathogenesis of *S. suis* in a piglet model [11]. However, *fhbp* expression was shown to be upregulated in brain and lungs during experimental infection of pigs with *S. suis* [12].

In order to evaluate the individual or combined contribution of Fhb, Fhbp as well as the CPS in the ability of *S. suis* to bind factor H, we constructed single isogenic as well multiple knock-out mutants of *S. suis* serotype 2 deficient for the above surface constituents. Our results show that binding of factor H to the *S. suis* surface increases adhesion to both epithelial and endothelial cells. However, recruitment of factor H to the bacterial surface is multifactorial. 

## 2. Results

### 2.1. Mutant Characterization

**Figure 1:**
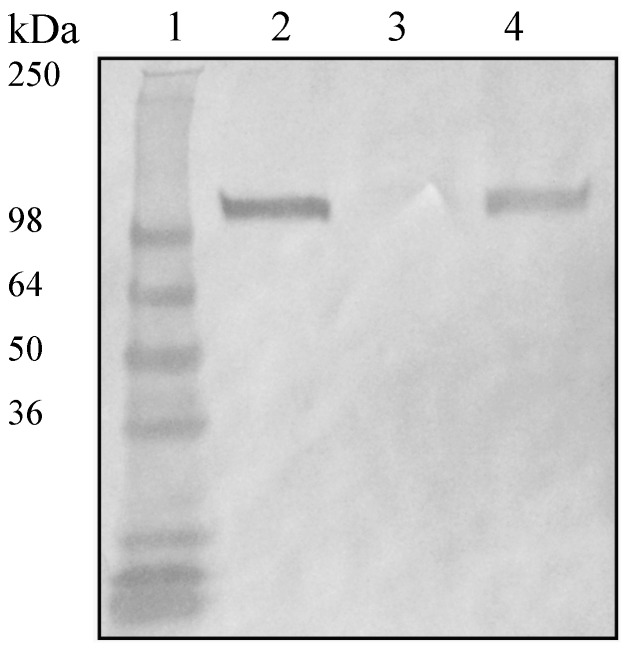
Western blot showing factor H binding protein (Fhbp) expression in *S. suis* wild-type strain P1/7 and complemented ∆*fhbp* mutant but not in the isogenic ∆*fhbp* mutant. Whole bacteria of *S. suis* wild-type strain P1/7 (lane 2), ∆*fhbp* mutant (lane 3) and complemented ∆*fhbp* mutant (lane 4) were tested for Fhbp expression. Samples were separated by SDS-polyacrylamide gel electrophoresis and transferred to a nitrocellulose membrane. Fhbp protein was detected with a monospecific rabbit polyclonal antiserum against Fhbp. Fhbp protein was not detected in ∆*fhbp* mutant, whereas a clear positive reaction was obtained for the wild-type strain and the complemented mutant. Molecular weights in kDa are indicated on the left side of the figure.

Inactivation of *fhb* and *cps* genes was previously done and mutants were already characterized; consequently, no further characterization of our equivalent mutants was done [11,13]. In order to further investigate the role of Fhbp in *S. suis* serotype 2, the gene encoding this surface protein [10] was inactivated by a specific in-frame allelic deletion in the wild-type strain P1/7. Western blotting using an anti-Fhbp polyclonal antibody depicted in Figure 1 clearly shows that the resulting Δ*fhbp* mutant does not express Fhbp (Figure 1). Complementation by the *fhbp* gene restored the expression of Fhbp. Inactivation of *fhbp* (or previously described inactivation of *fhb* and *cps2F*) had no major consequence on bacterial growth when compared to the wild-type strain (data not shown). Coagglutination and dot-ELISA tests showed that all mutants (with the exception of those including a *cps2F* deletion) were as encapsulated as the wild-type strain (results not shown). Consequently, hydrophobicity tests showed low hydrophobicity (<5%) for the wild-type strain as well as all mutants, except for those with the *cps2F* deletion (>90%).

### 2.2. Adhesion and Invasion Assays

**Figure 2:**
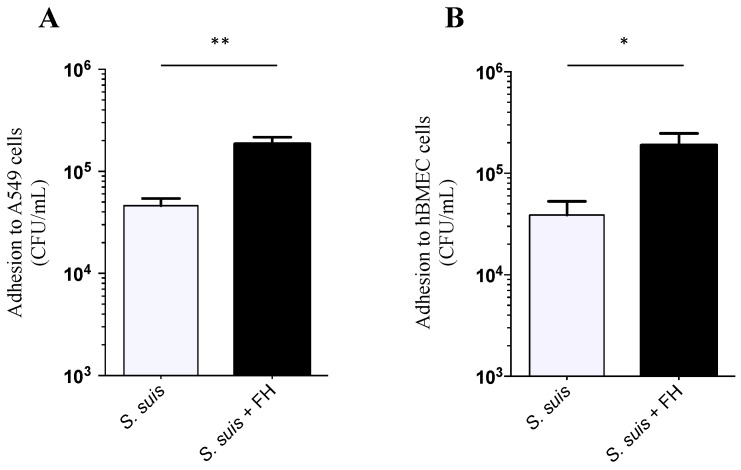

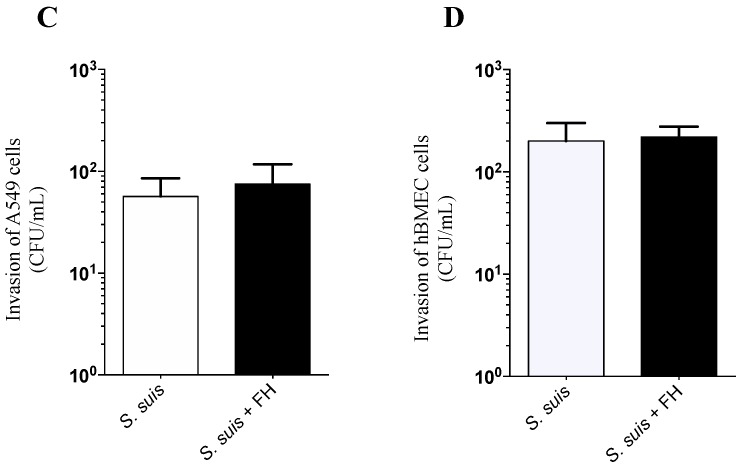
Effect of factor H on cell adhesion and invasion by *S. suis* wild-type strain P1/7. *S. suis* adhesion to (**A**) human lung epithelial cells A549 and (**B**) human brain microvascular endothelial cells (hBMEC). Results were determined after 1 h exposure of A549 and hBMEC cells to *S. suis*, followed by extensive washing of non-adherent bacteria and cell lysis to obtain *S. suis* viable counts. Results are expressed as recovered CFU/mL. Significant differences between the wild-type strain P1/7 preincubated with factor H and the same strain preincubated in phosphate buffered saline (PBS) were observed for both A549 and hBMEC cells (** *p* = 0.006 for A549 and * *p* = 0.04 for hBMEC), as determined by one-way ANOVA. *S. suis* invasion of (**C**) human lung epithelial cells A549 and (**D**) hBMEC. Results were determined after 1 h exposure of cells to *S. suis*, followed by antibiotic treatment to kill extracellular bacteria and by cell lysis to obtain *S. suis* viable counts. No significant differences were observed. Data are expressed as mean ± standard error of mean (SEM) of at least four independent experiments.

**Figure 3:**
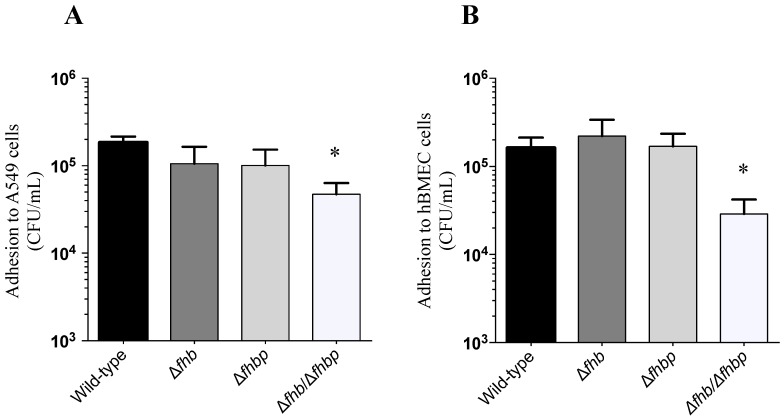

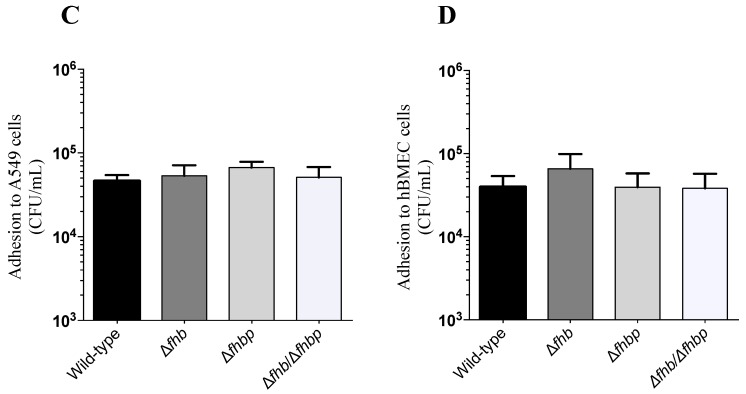
Effect of the deletion of *fhb* and *fhbp* on the *S. suis* adhesion to A549 and hBMEC cells in the presence or absence of factor H. Adhesion of *S. suis* factor H binding (Fhb) and Fhbp deficient mutants to (**A**,**C**) human lung epithelial cells A549 and to (**B**,**D**) hBMEC in presence (**A,B**) or absence (**C,D**) of human factor H. Experiments were performed as described in Figure 2. Results are expressed as recovered CFU/mL. Significant differences between the double knock-out ∆*fhb/*∆*fhbp* mutant and wild-type strain P1/7 as well as single mutants were observed in presence of factor H for both A549 (* *p* = 0.0279) and hBMEC cells (* *p* = 0.0214), as determined by one-way ANOVA. No significant differences were observed between the wild-type strain P1/7 and single deletion mutants (∆*fhb* and ∆*fhbp*). Data are expressed as mean ± SEM of at least four independent experiments.

The interactions (adhesion and invasion) of the *S. suis* wild-type strain (P1/7) as well as mutants defective for factor H binding proteins with human epithelial cells (A549) and human endothelial cells (hBMEC) in presence or absence of human factor H was investigated. As shown in Figure 2A,B, adhesion of *S. suis* wild-type strain to A549 human epithelial and hBMEC human endothelial cells was significantly increased in presence of factor H, especially for the epithelial cells (*p* = 0.006 and *p* = 0.04, respectively). In contrast, no significant differences were observed regarding invasion of both cell types (Figure 2C,D). The role of different factor H binding proteins in such interactions was further investigated. Adhesion levels to hBMEC and A549 cells of single knock-out Δ*fhb* and Δ*fhbp* mutants in the presence of factor H were similar to those obtained with the wild-type strain P1/7 (Figure 3A,B). The double knock-out Δ*fhb/*Δ*fhbp* mutant showed a significant decrease in adhesion levels to epithelial cells (*p* = 0.0279) and to endothelial cells (*p* = 0.0214) (Figure 3A,B). It has to be noted that the double knock out mutant adhered similarly to the wild-type strain to both cell types in absence of factor H (*p* > 0.05, Figure 3C,D).

### 2.3. Evaluation of the Role of Factor H Binding Proteins on *S. suis* Factor H Deposition

**Figure 4:**
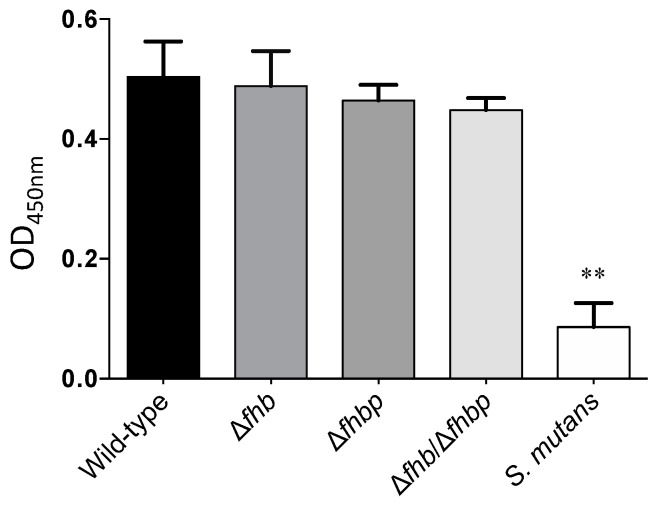
Deposition of factor H to the *S. suis* cell surface: role of Fhb and Fhbp. Deposition of factor H to the bacterial cell surface was detected using an ELISA assay. *Streptococcus mutans* was included as a negative control for factor H binding. There were statistically significant differences between all *S. suis* strains and *S. mutans* as determined by one-way ANOVA (** *p* < 0.01). No significant differences were observed between the *S. suis* wild-type strain P1/7 and isogenic mutants ∆*fhb*, ∆*fhbp* and ∆*fhb*/∆*fhbp*.

The deposition of factor H to *S. suis* strains was further evaluated. *Streptococcus mutans* was used as a negative control and showed only a weak deposition of factor H. Surprisingly, no significant differences were observed between the wild-type and *S. suis* mutant strains as depicted in Figure 4. Indeed, they were all similarly able to bind factor H (*p* > 0.05).

### 2.4. Evaluation of the Role of CPS for *S. suis* Factor H Deposition

**Figure 5:**
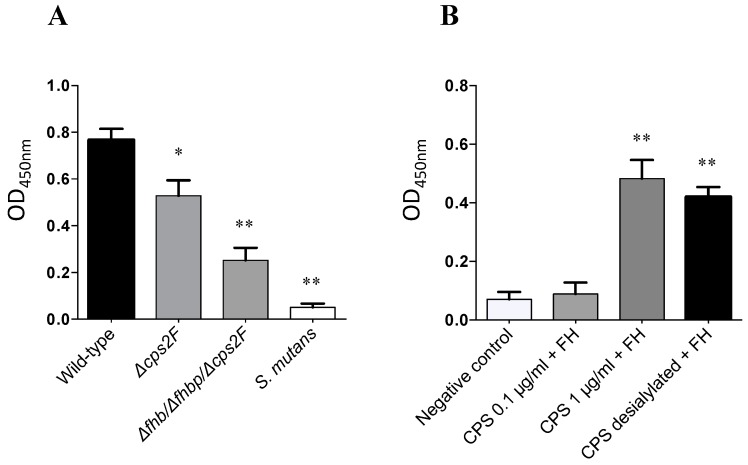
Deposition of factor H to the *S. suis* cell surface: role of capsular polysaccharide (CPS) and its sialic acid moiety. Results of ELISA showing binding of factor H to (**A**) non-encapsulated *S. suis* and to (**B**) *S. suis* purified CPS. There were statistically significant differences between groups for Figure 5A,B as determined by one-way ANOVA. In Figure 5A, significant differences with the wild-type strain are depicted with asterisks (* *p* < 0.05, ** *p* < 0.01). Data are expressed as mean ± SEM of at least three independent experiments. In Figure 5B, different concentrations (0.1 and 1 μg/mL) of precoated purified *S. suis* native and desialylated CPS were incubated with factor H (10 μg/mL). Significant differences were observed with factor H incubated with native and desialylated CPS at 1 μg/mL vs. control incubated without CPS (** *p* = 0.0006 and ** *p* = 0.0012, respectively). No significant differences were observed between native and desialylated CPS in their capacity to bind factor H (*p* > 0.05).

Aforementioned results suggest the presence of additional factors involved in factor H recruitment to the surface of *S. suis*, we further evaluated whether the CPS is also able to bind factor H at the bacterial surface. The recruitment of factor H in the absence of CPS was investigated using the non-encapsulated Δ*cps2F* and and the triple Δ*fhb*/Δ*fhbp*/Δ*cps2F* mutants. As shown in Figure 5A, the Δ*cps2F* mutant was significantly affected in its capacity to recruit factor H when compared to the wild-type strain (*p* = 0.0376). Moreover, the additional effect of CPS and factor H binding proteins can be observed with the triple mutant (Δ*fhb*/Δ*fhbp*/Δ*cps2F*), for which significant differences were observed when compared not only to the wild-type strain (*p* = 0.0004) but also to the Δ*cps2F* mutant (*p* = 0.0181).

To confirm that the CPS is able to bind factor H, and to evaluate the importance of the sialic acid moiety in such an interaction, an ELISA test using highly purified native and desialylated CPS was performed. Both native and desialylated CPS (at a concentration of at least 1 µg/mL) could significantly bind factor H (*p* = 0.0006 and 0.0012, respectively) (Figure 5B).

### 2.5. C3b Cleavage Assay

**Figure 6:**
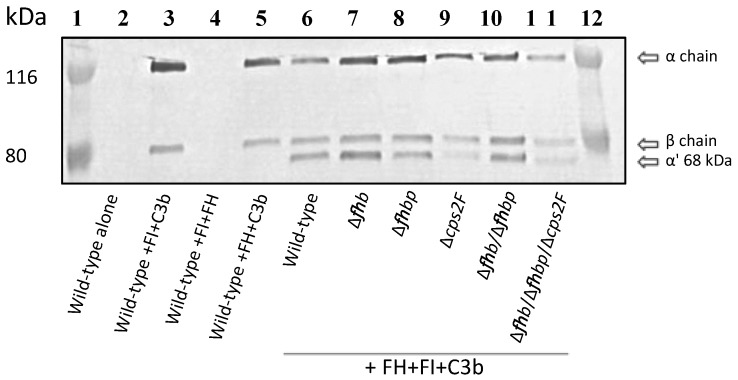
Factor-I cofactor assay showing C3b degradation by *S. suis* strains. Immunoblot shows that factor H bound to *S. suis* strains serves as cofactor for factor I (FI)-mediated cleavage of C3b, resulting in formation of an α’68 kDa chain. Lane: 1, molecular mass marker; 2, Wild-type strain P1/7 alone; 3, Wild-type strain P1/7 + FI + C3b; 4, Wild-type strain P1/7 + FI + FH; 5, Wild-type strain P1/7 + FH + C3b; 6, Wild-type strain P1/7 + FH + FI + C3b; 7, Δ*fhb* mutant strain + FH + FI + C3b; 8, Δ*fhbp* mutant strain + FH + FI + C3b; 9, Δ*fhb/*Δ*fhbp* mutant strain + FH + FI + C3b; 10, Δ*cps2F* mutant strain + FH + FI + C3b; 11, Δ*fhb*/Δ*fhbp*/Δ*cps2F* mutant strain + FH + FI + C3b; and 12, molecular mass marker. All strains retained the capacity to bound factor H in a way that serves as cofactor for factor I-mediated cleavage.

The functional activity of factor H bound to the surface of *S. suis* was tested using a factor I–cofactor assay. C3b degradation products were identified by SDS-PAGE/Western blot. As depicted in Figure 6, all strains tested retained the capacity to degrade C3b as shown by the appearance of the α’ 68 kDa cleavage product in addition to the α-chain (116 kDa) and the β-chain (75 kDa) of C3b. Positive control with wild-type strain P1/7 (lane 6) shows normal C3b cleavage product (α’ 68 kDa) by factor I in presence of factor H. The 68 kDa band does not appear with the wild-type strain P1/7 in the absence of factor H (lane 3; negative control).

### 2.6. Phagocytosis Assay

**Figure 7:**
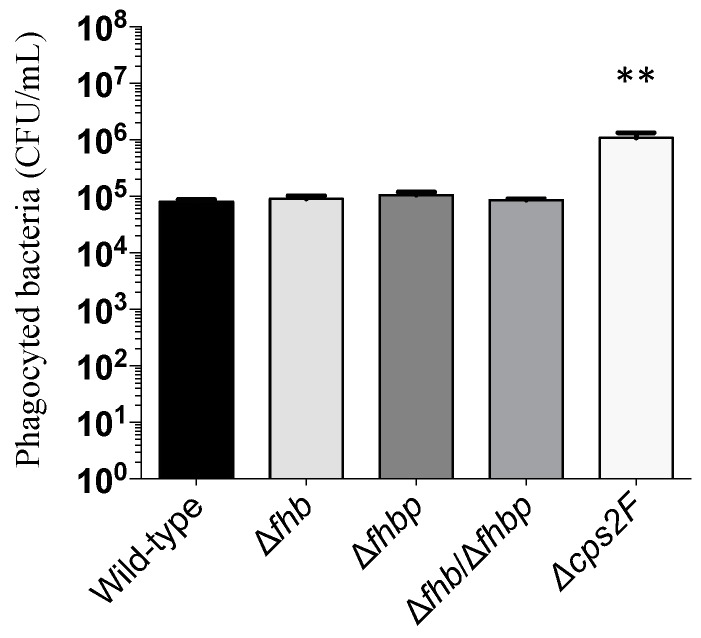
Phagocytosis of *S. suis* strains by THP-1 human macrophages in presence of complement-rich serum. Bacteria (1 × 10^7^ CFU/mL) were incubated for 90 min with cells (MOI = 100) in presence of human serum, followed by gentamicin/penicillin G treatment to kill any remaining extracellular bacteria after incubation. Intracellular counts were done after three washes and cell lysis with water. Results represent the mean (CFU/mL) ± SEM of four independent experiments. There were no statistical differences between the *S. suis* wild-type and any of the factor H-binding protein mutants. The non-encapsulated mutant (positive control) was significantly more phagocytosed as determined by one-way ANOVA (** *p* < 0.01).

We investigated the role of both Fhb and Fhbp on the resistance capacity of *S. suis* to complement-mediated opsonophagocytosis. As shown in Figure 7, no significant differences were observed when comparing the wild-type strain with the single or double Δ*fhb*/Δ*fhbp* mutant. In contrast, the non-encapsulated strain Δ*cps2F*, used as control, was susceptible to phagocytosis by macrophages showing significant difference with both wild-type and mutants strains (*p* < 0.0001) (Figure 7).

### 2.7. Whole Blood Bacterial Killing Assay

**Figure 8:**
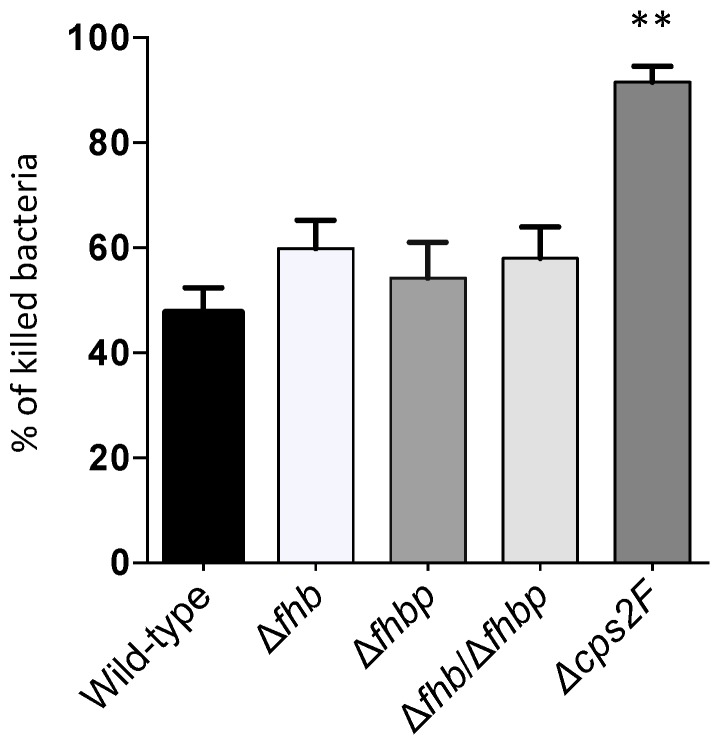
Killing of *S. suis* by swine whole blood cells. Bacteria (5 × 10^5^ CFU) were incubated for 120 min with swine whole blood or with blood serum (bacteria alone). The percentage of killed bacteria was calculated as follows: 1 – (Bacteria recovered in blood/bacteria recovered in serum) × 100%. Data are expressed as mean ± SEM of at least three independent experiments. There were not statistical differences between the *S. suis* wild-type and any of the factor H-binding protein mutants. The non-encapsulated mutant (positive control) was significantly more killed as determined by one-way ANOVA (** *p* < 0.01).

Bacterial killing by whole swine blood showed no differences between the wild-type strain P1/7 and either of the Δ*fhb*, Δ*fhbp* or Δ*fhb*/Δ*fhbp* mutants (Figure 8). Percentage of killing varied from 40 to 60% for all strains. Only the non-encapsulated Δ*cps2F* mutant strain was shown to be highly susceptible, as expected, with 90% of bacteria being killed (Figure 8).

## 3. Discussion

The role of complement during the systemic infection caused by *S. suis* is still controversial. In an early report, Brazeau et al. suggested a limited role of complement in phagocytosis and killing of well encapsulated *S. suis* [14]. More recent studies showed that the complement limits *S. suis* invasion when using an intranasal mouse model of infection [15]. However, the role of complement in the *S. suis* systemic infection still remains to be confirmed.

Factor H is the key fluid phase regulator of the alternative complement pathway and acts as a cofactor in the factor I-mediated proteolysis of C3b [16]. Hence, pathogenic bacteria have developed mechanisms to recruit factor H to their surface to avoid complement attack and opsonophagocytosis. Indeed, several pathogens possess adhesins that are able to recruit factor H to their surface to degrade C3b in iC3b in order to reduce opsonophagocytosis by host cells [17,18]. Recruitment of factor H may also help bacteria to adhere and to invade epithelial and endothelial cells [19]. For most pathogens, different proteins expressed at the bacterial surface are responsible for factor H deposition [20]. In the case of *S. suis*, two factor H-binding proteins (Fhb and Fhbp) have been reported and characterized so far [10,11]. In the present study, we investigated the role of factor H, as well as those played by Fhb and Fhbp, as adhesion/invasion molecules for *S. suis* during bacterial interaction with host cells. In addition to these proteins, the role of CPS in the recruitment of factor H was also studied.

Results showed that factor H acts as an enhancing adhesion molecule for *S. suis* to epithelial and, to a lesser extent, endothelial cells. This function had been previously described for other human pathogens, including *S. pneumoniae* [19]. It has been suggested that factor H binds to pneumococci, and bound factor H is oriented in a way that it can interact with polyanionic molecules (glycoamynoglycans) on the surface of host cells [19]. Receptors used by the factor H bound to the *S. suis* surface in order to promote bacterial adhesion are still unknown. The fact that the increased adhesion was considerably more significant with epithelial cells may indicate that this mechanism could mainly benefits bacteria during the early steps of the infection. Interestingly, although single Δ*fhb* and Δ*fhbp* mutants adhered similarly to the wild-type strain, a slight but significant reduction in adhesion to both cell types was observed with the double Δ*fhb*/Δ*fhbp*, suggesting, to a certain extent, an additive role when the two proteins are present. However, the possibility that the inactivation of one *S. suis* factor H binding protein (Fhb or Fhbp) induces the overexpression of the second protein (Fhbp or Fhb, respectively) cannot be ruled out. The actual role in vivo of factor H-dependent increased adhesion should be confirmed. In contrast to what has been described for *S. pneumoniae* [19], factor H does not increase the invasion properties of *S. suis* to the epithelial or endothelial cells tested. As expected, the absence of Fhb and Fhbp did not influence host cell invasion.

Interestingly, the bacterial proteins evaluated in this study do not play a critical role in adhesion/invasion to cells per se, since Δ*fhb*, Δ*fhbp* single mutants as well as the double Δ*fhb*/Δ*fhbp* mutant behave similarly to the wild-type strain in the absence of factor H. These results were unexpected, since the pneumococcal surface protein C (PspC), which presents homology to the *S. suis* Fhbp, directly participates in *S. pneumoniae* cell adhesion by binding to host glycoconjugates and sialic acid residues [21]. In addition, the Fhb protein has been reported to be responsible for the *S. suis* binding to Galα1-4Gal present in glycolipid Gb03—abundant in epithelial and endothelial cells [22,23]. We could not find any previous work indicating that the Gb03 is precisely present in the epithelial cell line A549. However, this glycolipid has previously been shown to be present in the hBMEC line used in this study [24]. If the factor H-binding proteins of *S. suis* do not play any role in adhesion to epithelial and endothelial cells or if such a hypothetical role is redundant due to the presence of additional adhesins remains to be studied.

Surprisingly, although both Fhb and Fhbp proteins together play a limited but certain role in factor H-mediated adhesion to cells together, the absence of either one or both of these proteins had no effect on the overall capacity of *S. suis* to recruit factor H at the bacterial surface. It might be possible that, although similar amounts of factor H may be recruited to the bacterial surface by other factor H binding factors, specific binding of factor H to both studied proteins may induce steric modifications that could slightly modify the enhanced capacity of bacteria to adhere to host cells. It has been previously shown that very specific domains of factor H are involved in the interactions with factor H-binding proteins [25]. However, this hypothesis remains to be elucidated.

Since factor H is still recruited at the bacterial surface of factor H-binding protein defective mutants, we hypothesized that additional bacterial factors may play important roles in such activity. It has been reported that some sialylated pathogens are able to recruit factor H due to the presence of sialic acid at their surface, as it is the case of *Neisseria gonorrhoeae* and *Pasteurella aeruginosa* [26,27]. Since the CPS of *S. suis* serotype 2 is rich in sialic acid [28], the capacity of such a CPS to interact with factor H was evaluated. To carry out such studies, a capsule-deficient (Δ*cps2F*) mutant and a triple mutant deficient in both factor H binding proteins and the CPS (Δ*fhb/*Δ*fhbp/*Δ*cps2F*) were produced. Interestingly, the non-encapsulated mutant recruited significantly less factor H to the bacterial surface than the wild-type strain. Although the CPS is thought to limit the exposition of cell surface components [29], it does not seem to be the case for factor H binding proteins. It has also been suggested for other pathogens that the presence of CPS does not reduce surface availability of factor H binding proteins [30,31]. Indeed, a clear collaborative and additive factor H-recruitment activity between Fhb and Fhbp proteins together with the CPS can be suggested, since the triple mutant (Δ*fhb/*Δ*fhbp/*Δ*cps2F*) recruited significantly less factor H deposition at the surface than both, the wild-type and the double-mutant (Δ*fhb/*Δ*fhbp*) mutant.

Marques et al. (1992) demonstrated that wild-type Group B *Streptococcus* binds lower levels of active C3b as compared to mutants deficient in capsule and sialic acid expression, postulating that this could be due to the binding of factor H to sialic acid [32]. However, the sialic acid mutant used in that particular study was poorly encapsulated [33] and differences observed could have been the result of the absence of sialic acid, CPS or both. A function of the CPS in resistance to complement deposition has already been shown for *S. suis* [13]. In addition, it has been recently demonstrated that the CPS would play an important role in the protection against the complement system in a *S. suis* experimental mucosal infection model [15]. In this study, and for the first time, we report that the CPS from a Gram-positive pathogen is able to recruit factor H. Desialylated CPS was, unexpectedly, also able to bind similar amounts of factor H than the native CPS, indicating that sialic acid is not the main sugar involved in binding such host factor. An encapsulated but sialic-acid negative mutant cannot be used to confirm these results, since such mutants are either non-encapsulated [33,34] or lethal [35] for *S. suis*. The mechanisms by which the CPS binds the factor H, as well as the ability of CPS from different *S. suis* serotypes to bind factor H remain to be elucidated. It has been previously shown that different serotypes of *S. pneumoniae* were able to differently recruit human factor H to their cell surface [30], although the specific role of the CPS was never investigated. Finally, the role of factor H interactions with a non-encapsulated *S. suis* strain on bacterial adhesion studies could not be performed, since capsule deficient mutants already adhere at very high levels to cells [36].

As mentioned, the triple (Δ*fhb/*Δ*fhbp/*Δ*cps2F*) mutant is still able to bind factor H to a certain extent, as compared to the *S. mutans* used as negative control. In addition, the wild-type strain of *S. suis* as well as all mutant strains tested in the factor-I cofactor assay equally retained the capacity to degrade C3b in the presence of factor H. These results strongly suggest the presence of additional *S. suis* surface components able to recruit factor H. For example, Shao et al. (2011) identified an immunogenic cell surface-associated protein (histidine triad protein of *S. suis*) involved in evasion of complement-mediated host innate immune responses by preventing C3 deposition on the bacterial surface of *S. suis* by so far unknown mechanisms [37]. Hence, other proteins redundantly being able to bind factor H may also exists.

Results obtained with the factor-I cofactor assay were confirmed by phagocytosis and killing assays. Single as well as the double Δ*fhb/*Δ*fhbp* mutants were as resistant to phagocytosis as the wild-type strain. This is the first time that the Fhbp is evaluated. However, these results are in disagreement with those previously published with a Fhb deficient mutant derived from a highly virulent Chinese strain [11], where the authors showed that the Fhb mutant was highly susceptible to phagocytosis by human neutrophils [11]. In addition, although that study also reported that the Δ*fhb* mutant was sensitive to the blood-killing test and was less virulent in pigs (with lower levels of bacteremia) [11], no differences could be observed with the whole cell blood-killing test in the present study. Besides some technical details in the methodology as well as the use of different strains, we do not have clear explanations to explain such differences; in our hands, both Fhb and Fhbp proteins do not seem to be highly involved in factor H recruitment, resistance to phagocytosis or killing, and it would be highly surprising that these proteins play a critical role in virulence. In fact, single- and double-factor H binding proteins mutants used in the present study were shown to be as virulent as the wild-type strain in a mouse model of infection (unpublished). Finally, the role of the CPS in phagocytosis and killing has largely been shown [13,34], and it is probably not linked to the factor H recruitment activity.

In conclusion, binding of factor H to the *S. suis* surface increases bacterial adhesion to host cells, especially epithelial cells, and allows degradation of C3b. This factor H recruitment seems to modestly occur through Fhb, Fhbp and the CPS. However, these factors do not seem to be critical for such factor H binding activity as well as its consequences. It seems that recruitment of factor H to *S. suis* surface is multifactorial and redundant. 

## 4. Materials and Methods

### 4.1. Bacterial Strains and Culture Conditions

The virulent serotype 2 strain P1/7 was used as the wild-type strain for in-frame allelic deletion mutagenesis. *Streptococcus mutans* strain 25175 (ATCC, Manassas, VA, USA) was used as a negative control for factor H deposition studies. Bacterial strains and plasmids used in this study are listed in Table 1. *Streptococcus* strains were grown in Todd-Hewitt broth (THB) or agar (THA) (Becton-Dicksinson, Sparks, MD, USA) at 37 °C. *Escherichia coli* strains were grown in Luria-Bertani (LB) broth or agar (Becton-Dickinson) at 37 °C. When needed, antibiotics (Sigma-Aldrich Canada Co., Oakville, ON, Canada) were added to the culture media at the following concentrations: for *S. suis*, spectinomycin (Sp) at 100 µg/mL; for *E. coli*: kanamycin (Km) and Sp at 50 µg/mL; and ampicillin at 100 µg/mL. 

### 4.2. Cell Lines and Cell Culture

The human lung epithelial cell line A549 (ATCC CCL-185) was used and cultured until confluent as previously described [19]. The human brain microvascular endothelial cell line (hBMEC, gift from Dr. K. S. Kim, Johns Hopkins University School of Medicine, MD, USA) was used and cultured until confluent as previously described [42]. THP-1 human monocytic cell line (ATCC TIB-202) was used and cultured as previously described [43].

### 4.3. DNA Manipulations

*S. suis* genomic DNA was purified using InstaGene Matrix solution (BioRad Laboratories, Hercules, CA, USA). Oligonucleotide primers used in this study are listed in Table 2. Transformations of *Escherichia coli* were performed following the manufacturers’ recommendations (Invitrogen, Burlington, ON, Canada). Extraction and purification of recombinant plasmids were performed using QIAprep Spin Miniprep kit (Qiagen Valencia, CA, USA). Restriction enzymes and DNA-modifying enzymes were purchased from Fisher Scientific (Ottawa, ON, Canada) and used according to the manufacturers’ recommendations. PCR reactions were carried out with an iProof high-fidelity DNA polymerase (BioRad Laboratories) or with Taq DNA polymerase (Qiagen). Oligonucleotide primers were ordered from Integrated DNA Technologies (IDT, Coralville, IA, USA). Amplification products were purified using the QIAgen PCR purification kit (Qiagen) and sequenced with an ABI 310 automated DNA sequencer using the ABI PRISM dye terminator cycle sequencing kit (Applied Biosystems, Foster City, CA, USA).

### 4.4. Construction of Allelic Deletion Mutants

Fhb protein and CPS deficient mutants (∆*fhb* and ∆*cps2F*) have been previously obtained and characterized [11,13]. Fhbp, double Fhb/Fhbp and triple Fhb/Fhbp/CPS deletion mutants were obtained for the first time in the present study. Precise in-frame deletions were achieved using splicing-by-overlap-extension PCR [44]. Overlapping PCR-products generated by PCR were cloned into plasmid pCR2.1 (Invitrogen), extracted using EcoRI, and cloned into the thermosensitive *E. coli*-*S. suis* shuttle vector pSET4s previously digested with EcoRI, giving rise to the p4∆*fhb*, p4∆*fhbp* and p4∆*cps2F* mutation vectors. Final constructions of pSET4s vector were electroporated into *S. suis* competent cells with a Biorad Gene Pulser Xcell apparatus (BioRad Laboratories) under specific conditions (12.5 kV/cm, 200 Ω and 25 uF) and cells were plated on THA supplemented with Sp (THA + SP) and incubated for 3 days at 28 °C. Several Sp-resistant colonies were then subcultured again on THA + SP for 3 days at 28 °C. The candidates were next cultured on THA + SP and incubated at 37 °C for two successive passages and then screened for first crossing-over event. Lost of vector was induced by incubation of candidates at 28 °C. Temperature- and Sp-resistant clones were successively cultured on THA and THA + SP to obtain Sp-sensitive candidates. Deletion of the genes was confirmed by PCR and sequence analysis.

The lack of expression of Fhbp in the ∆*fhbp* mutant was evaluated by immunoblot. Briefly, bacteria from 10 mL of overnight cultures of the wild-type, mutant and complemented strains under investigation were harvested by centrifugation and resuspended in PBS at OD_600_ = 0.600. Ten microliters of bacteria were mixed with 10 µL of denaturing electrophoresis buffer and boiled for 10 min. Samples were then electrophoresed on sodium dodecyl sulphate-polyacrylamide (SDS) gels. Western blotting was carried out as previously described by Burnette [45], using antisera from immunized rabbit with recombinant Fhbp protein, which was cloned, expressed and purified as previously described [10].

The presence of the CPS in ∆*fhb*, ∆*fhbp* and ∆*cps2F* mutants were tested by the coagglutination and dot-ELISA tests using anti-serotype 2 polyclonal and monoclonal antibodies, respectively [46,47]. In addition, surface hydrophobicity was tested as previously described [34].

### 4.5. Construction of Complemented *Δfhbp* Mutant

Complemented ∆*fhb* and ∆*cps2F* mutants had previously been done [11,13]. Since the Fhbp deficient mutant was obtained for the first time in this study, a complemented strain was also produced. Intact *fhbp* gene was amplified from genomic DNA of the wild-type strain with primers containing specific restriction sites (Table 2). PCR products and pMX1 vectors were then digested with the appropriate restriction enzyme before ligation. Final constructions were cloned into *E. coli* MC1061. Plasmid pMX1 is a derivative of the *S. suis*–*E. coli* shuttle cloning vector pSET2 and possesses the *S. suis* malX promoter for transgene expression in *S. suis* [40]. Complementation of Δ*fhbp* mutant was achieved by transformation with pMXfhbp by electroporation under the same aforementioned conditions. Presence of the plasmid within the mutant was confirmed by PCR. Expression of the Fhbp was studied as described above.

### 4.6. Adhesion and Invasion Assays

The A549 and hBMEC cells were used to evaluate the adhesion properties of the different mutants and wild-type strains in presence or absence of factor H. Bacteria were grown to mid-logarithmic phase, harvested by centrifugation, washed three times and resuspended in PBS at OD_600_ = 0.600. One-hundred microliters of bacterial suspension (equivalent to 1 × 10^7^ CFU) were preincubated with 2 µg of human factor H (Quidel, San Diago, CA, USA) for 20 min at 37 °C. Four-hundred microliters of RPMI were then added to bacteria without washing out unbound factor H, as previously described for other streptococci [19]. Cells were infected with *S. suis* strains (1 × 10^7^ CFU/well, multiplicity of infection (MOI): 50) and incubated at 37 °C in 5% CO_2_ for 1 hour. An adhesion assay—which in fact quantifies total intracellular bacteria and surface-adherent bacteria—was performed as previously described [48]. Cell monolayers were washed five times with PBS and lysed with sterile cold water. Viable bacteria were determined by plating samples onto THA using an Autoplate^®^ 4000 Automated Spiral Plater (Spiral Biotech, Norwood, MA, USA). The invasion assay was performed using the antibiotic protection assay as previously described [48]. Briefly, after the initial incubation time, cell monolayers were washed twice with PBS and incubated for an additional 1 h with medium containing 5 µg/mL penicillin G (Sigma) and 100 µg/mL of gentamicin (Gibco, Burlington, ON, Canada) in order to kill extracellular bacteria. Cell monolayers were then washed three times with PBS and lysed with sterile cold water. Viable intracellular bacteria were determined as described above. Optimal incubation time and MOI for both adhesion and invasion tests were chosen based on published studies [42,49] and preliminary tests done with the wild-type strain. Adhesion and invasion tests were done in duplicates and repeated at least four times in independent experiments.

### 4.7. Evaluation of Factor H Deposition to *S. suis* Strains

Cell-based enzyme-linked immunosorbent assay (ELISA) was used to evaluate the factor H deposition at the bacterial surface of different mutants obtained in this study, as previously described with some modifications [10]. Briefly, washed harvested bacteria were adjusted to an OD_600_ of 0.2 in 50 mM carbonate buffer (pH 9.6). One-hundred microliters per well were added to flat-bottom 96-well microplates (Nunc-immuno^®^ Polysorp; Nalge Nunc International, Rochester, NY, USA) and incubated for 2 h at room temperature. Bacterial suspension was removed and bound bacteria were fixed with glutaraldehyde (0.05%) for 45 min. Wells were then washed three times with PBS-Tween 0.05% (PBS-T) and blocked with PBS-T supplemented with 2% fat-free milk for 1 hour. After washing, fixed bacteria were then incubated in presence of 100 µL of human factor H (10 µg/mL in PBS) for 2 hours at 37 °C. Plates were then washed three times to remove unbound factor H. The deposition of factor H at the bacterial surface was detected by goat antisera against human factor H (Quidel) and HRP-conjugated donkey anti-goat IgG antibody (Jackson ImmunoResearch, West Grove, PA, USA). The OD_450_ was recorded with a microplate reader after adding horseradish peroxydase substrate. Each assay was repeated in duplicates in four independent experiments.

### 4.8. Factor H Recruitment by the Capsular Polysaccharide

In order to investigate the potential role of the CPS of *S. suis* serotype 2 as well as its sialic acid component in factor H recruitment, an ELISA was performed as previously described [50], with some modifications. Briefly, 100 µL of native or desialylated CPS (0.1 or 1 µg/mL in carbonate buffer), highly purified as previously described [28], was used to coat wells of Polysorp flat-bottom 96-well microplate and incubated at 37 °C for 2 h. Unbound CPS was washed with PBS-T three times and wells were blocked as described above. After washing, purified factor H (10 µg/mL, chosen based on preliminary dose-response studies; not shown) was added to the wells and incubated for 2 h at room temperature. Correct binding of purified CPS to the wells was verified as described [50]. Deposition of factor H was detected as described above.

### 4.9. C3b Cleavage Assay

The functional activity of factor H bound to *S. suis* was assayed using a factor I–cofactor assay as described by Vaillancourt et al. [10]. Briefly, *S. suis* cells were incubated with human factor H (0.7 µg/mL) for 2 h at 37 °C. Bacteria were then washed three times in PBS and suspended in the same buffer, and human C3b (4.5 μg/mL; Calbiochem EMD chemicals, Billerica, MA, USA) as well as human factor I (2.5 μg/ mL; Quidel) were added. Following incubation at 37 °C for 2 h, denaturing electrophoresis buffer was added and the mixture was subjected to SDS-10% PAGE. Proteins were electrophoretically transferred onto a nitrocellulose membrane and C3b degradation products were visualized by Western blotting using goat anti-human C3b (1:500; Quidel) and then AP-conjugated mouse anti-goat IgG antibody (1:1000; Santa Cruz Biotechnology, Dallas, TX, USA). Bands were revealed by adding the AP substrate.

### 4.10. Phagocytosis Assays

Activation of THP-1 monocytes was carried out as described by Segura et al. with some modifications [43]. Briefly, differentiation of monocytes was carried out by pre-treatment with phorbol 12-myristate 13-acetate (Sigma) (100 ng/mL) for 48 h prior to the test. Following differentiation, cells were washed three times with PBS and medium without antibiotics was added to each well. Bacteria were grown to mid-logarithmic phase and harvested by centrifugation. After incubation, 450 µL of freshly thawed human serum (complement preserved, Quidel) were added to bacteria. Cells were infected with bacterial preparation (1 × 10^7^ CFU/well, MOI: 10) and incubated at 37 °C in 5% CO_2_ for 90 min. Optimal incubation time and MOI were chosen based on preliminary studies (data not shown). After incubation, cell monolayers were washed twice with PBS and incubated for 1 h with medium containing 5 µg/mL penicillin G (Sigma) and 100 µg/mL of gentamicin (Gibco) to kill extracellular bacteria. Cell monolayers were washed three times with PBS and lysed with sterile cold water. Viable intracellular bacteria were determined by plating serial dilutions on THA as described above. Each test was repeated twice in three independent experiments.

### 4.11. Whole Blood Bacterial Killing Assay

Whole blood bacterial killing assay was adapted from whole blood culture system as previously described [51]. Blood from three 5-week-old healthy pigs was collected from the jugular vein using Vacutainer Heparin blood collection tubes. Animals originated from a specific pathogen-free herd, which had not presented any isolation of *S. suis* from diseased animals for at least the last 2 years. Blood was then diluted in RPMI 1640 culture medium (Invitrogen, Burlington, ON, Canada) in order to obtain a concentration of 1 × 10^7^ leucocytes/mL. Plasma was used as control and similarly processed as the blood sample. Bacteria were grown and washed as described for phagocytosis assays and suspended in RPMI 1640 medium to a concentration of 1 × 10^6^ CFU/mL. For killing assay, 250 µL of bacterial preparation were then added to 250 µL of blood mixture. Infected blood cells and reference control (plasma) samples were collected after 2 h of incubation at 37 °C in 5% CO_2_ with manual agitation every 20 min. To determine bacterial counts, serial dilutions of infected blood cultures were plated onto THB agar to accurately determine the CFU/mL. The percentage of killed bacteria was calculated as follows: 1 – (Bacteria recovered in blood/Bacteria in plasma) × 100%. Each test was repeated twice in three independent experiments.

### 4.12. Statistical Analysis

All data are expressed as mean ± SEM. Data were analyzed for significance using the one-way ANOVA test followed by a Dunnett post-hoc test for multiple comparisons or with Student’s *t*-test for comparisons between two groups. A *p* value < 0.05 was used as a threshold for significance (*). Values < 0.01 were considered as highly significant (**).

## 5. Conclusions

Results obtained in this study showed a role of factor H in the adhesion of *S. suis* to epithelial and, to a lesser extent, endothelial cells. Both Fhb and Fhbp proteins play a certain role in such increased factor H-dependent adhesion. None of these proteins were a critical adhesin per se (in the absence of factor H). The CPS, independently of the presence of its sialic acid moiety, was also shown to bind factor H. The absence of both factor H-binding proteins does not influence the resistance of *S. suis* to phagocytosis by human macrophages and to bacterial killing by swine blood. It seems that recruitment of factor H to *S. suis* surface is multifactorial and redundant.

## Figures and Tables

**Table 1:** Bacterial strains and plasmids used in this study.

Strains/Plasmid	General Characteristics	Source/Reference
*Escherichia coli*		
TOP 10	F-mrcA Δ(mrr-hsdRMS-mcrBC)ϕ80 lacZΔM5 ΔlacX74 recA1 araD139 Δ(ara-leu) 7697 galU galK rpsL (StrR) endA1 nupG	Invitrogen
MC1061	araD139 Δ(ara-leu)7697 ΔlacX74 galU galK hsdR2(rK-mK+) mcrB1 rpsL	[38]
*Streptococcus suis*		
P1/7	Wild-type strain, highly encapsulated serotype 2 strain isolated from a clinical swine case of infection in the United Kingdom	[39]
Δ*cps2F*	Non-encapsulated isogenic mutant strain derived from strain P1/7. Deletion of the *cps2F* gene	[13]
Δ*fhb*	Fhb expression-deficient strain derived from strain P1/7. Deletion of the *fhb* gene (SSU0253)	This work
Δ*fhbp*	Fhbp expression-deficient strain derived from strain P1/7. Deletion of the *fhbp* gene (SSU0186)	This work
Δ*fhb/*Δ*fhbp*	Fhb and Fhbp expression-deficient strain derived from strain P1/7. Deletion of the *fhb* and *fhbp* genes	This work
Δ*fhb/*Δ*fhbp/*Δ*cps2F*	Non-encapsulated mutant derived from strain Δ*fhb*/Δ*fhbp*. Deletion of the *cps2F* gene	This work
compΔ*fhbp*	Mutant Δ*fhbp* complemented with pMXfhbp complementation vector	This work
*Streptococcus mutans* 25175		
	Wild-type strain, isolated from a carious dentine case	ATCC 25175
Plasmids		
pCR2.1	Apr, Kmr, oriR(f1) MCS oriR (ColE1)	Invitrogen
pSET-4s	Thermosensitive vector for allelic replacement. Replication functions of pG+host3, MCS oriR pUC19 lacZ SpR	[40]
pMX1	Replication functions of pSSU1, MCS pUC19 lacZ SpR, malX promoter of *S. suis*, derivative of pSET2	[40,41]
p4Δ*fhb*	pSET-4s carrying the construct for *fhb* allelic replacement	This work
p4Δ*fhbp*	pSET-4s carrying the construct for *fhbp* allelic replacement	This work
p4Δ*cps2F*	pSET-4s carrying the construct for *cps2F* allelic replacement	This work
pMXfhbp	pMX1 complementation vector carrying intact *fhbp* gene	This work

**Table 2:** Oligonucleotide primers used in this study.

Oligonucleotide Primers, Sequence (5’–3’) ^a^		Constructs
fhbp-ID1	ACTGACAACATGACCGACCTCC	p4Δ*fhbp*
fhbp-ID2	TGTTGAAGTCTCTGTCGTCGC	p4Δ*fhbp*
fhbp-ID3	AAGTCATAAGGGCGCACCTTC	p4Δ*fhbp*
fhbp-ID4	TGTAGCCAGCGATAAGGCTCTG	p4Δ*fhbp*
fhbp-ID5	AACAGCCAGGCTTATGGAAGG	p4Δ*fhbp*
fhbp-ID6	TATAGCTGTAGCGACACGAATACTATATCT	p4Δ*fhbp*
fhbp-ID7	AGATATAGTATTCGTGTCGCTACAGCTATA	p4Δ*fhbp*
fhbp-ID8	TGTCAAGCCAATCCATGTCTGG	p4Δ*fhbp*
fhb-ID1	TCGGTGCTATCTTGCGTAGTC	p4Δ*fhb*
fhb-ID2	CATCTGGTTCTAGCGATTCTGC	p4Δ*fhb*
fhb-ID3	TGATGCCAAAAGCAGAGGCAC	p4Δ*fhb*
fhb-ID4	TGGAACTTTCGAGGTCGGTG	p4Δ*fhb*
fhb-ID5_EcoRI ^b^	GGCGCGAATTCCAAAGTTCTTGCCAGATGCCAC	p4Δ*fhb*
fhb-ID6	CCAGCCTATTGCGCTCCCTAATACGACTGT	p4Δ*fhb*
fhb-ID7	ACAGTCGTATTAGGGAGCGCAATAGGCTGG	p4Δ*fhb*
fhb-ID8_PstI ^b^	GGCGCCTGCAGAAATTTCCGCCCCTGACACAC	p4Δ*fhb*
pFHBP_F_PsI ^b^	GCGCCTGCAGCACATCCGACCACCTGAATATC	pMXfhbp
pFHBP_R_PstI ^b^	GGCGCCTGCAGGTTCTAAAAAGAGGCTGGGCG	pMXfhbp
cps-ID1	CCAGCAAAGTATGGTGGTTTCG	p4Δ*cps2F*
cps-ID2	GCGCACCAACTTCTCTTAATGC	p4Δ*cps2F*
cps-ID3	CTTAGTCACTCCGAACTCACCG	p4Δ*cps2F*
cps-ID4	CCACGCCAGATTCAATGAGC	p4Δ*cps2F*
cps-ID5	AGACGGTCATGAATGGCTACG	p4Δ*cps2F*
cps-ID6	GAGGGAGGTGTAGACTTCTGCTCCAGCATG	p4Δ*cps2F*
cps-ID7	CATGCTGGAGCAGAAGTCTACACCTCCCTC	p4Δ*cps2F*
cps-ID8	CATCAGAATGATGCCAAACAGG	p4Δ*cps2F*

^a^ Oligonucleotide primers were from IDT; ^b^ Restriction sites are underlined.

## Abbreviations

The following abbreviations are used in this manuscript:

CFU	colony-forming unit
CPS	capsular polysaccharide
Km	kanamycin
hBMEC	human brain microvascular endothelial cells
MOI	multiplicity of infection
PBS	phosphate-buffered saline
PBS-T	PBS-tween
SEM	standard error of the mean
Sp	spectinomycin
THA	Todd-Hewitt agar
THB	Todd-Hewitt broth
